# Long-GRIN-Lens Microendoscopy Enabled by Wavefront Shaping for a Biomedical Microdevice: An Analytical Investigation

**DOI:** 10.3390/ma14123392

**Published:** 2021-06-18

**Authors:** Guigen Liu, Jeon Woong Kang, Oliver Jonas

**Affiliations:** 1 Department of Radiology, Brigham and Women’s Hospital, Harvard Medical School, 221 Longwood Ave, Boston, MA 02115, USA; gliu19@bwh.harvard.edu; 2 Laser Biomedical Research Center, G. R. Harrison Spectroscopy Laboratory, Massachusetts Institute of Technology, Cambridge, MA 02139, USA; jwkang76@mit.edu

**Keywords:** GRIN-lens microendoscopy, two-photon imaging, wavefront shaping, biomedical microdevice, in vivo drug response testing

## Abstract

We analytically investigate the feasibility of long graded-index (GRIN)-lens-based microendoscopes through wavefront shaping. Following the very well-defined ray trajectories in a GRIN lens, mode-dependent phase delay is first determined. Then, the phase compensation needed for obtaining diffraction limited resolution is derived. Finally, the diffraction pattern of the lens output is computed using the Rayleigh–Sommerfeld diffraction theory. We show that diffraction-limited resolution is obtained for a 0.5 mm diameter lens with a length over 1 m. It is also demonstrated that different imaging working distances (WDs) can be realized by modifying the phase compensation. When a short design WD is used, a large imaging numerical aperture (NA) higher than 0.4 is achievable even when a low NA lens (NA = 0.1) is used. The long- and thin-GRIN-lens-based microendoscope investigated here, which is attractive for biomedical applications, is being prioritized for use in a clinical stage microdevice that measures three-dimensional drug responses inside the body. The advance described in this work may enable superior imaging capabilities in clinical applications in which long and flexible imaging probes are favored.

## 1. Introduction

**Figure 1:**
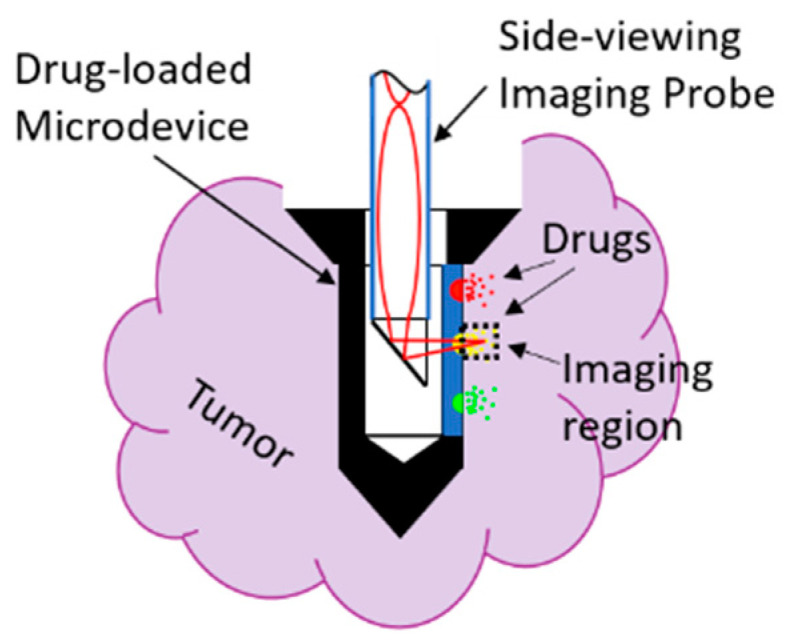
Schematic showing the implantable microdevice equipped with a side-viewing imaging probe.

An implantable in vivo biomedical microdevice [1] that combines drug delivery with optical imaging of tumor responses directly within the tumor has emerged as a powerful platform for high-throughput in situ screening of drugs in patients [2,3]. The microdevice enables evaluation of drug responses in their natural microenvironment, which is critical for efficient drug development and therapy selection for each individual patient. To maximize the impact of such microdevices, an in situ optical imaging probe capable of visualizing the drug responses in real time should be integrated (see Figure 1), especially in deep regions where long probes are required to circumvent the shallow penetration depth associated with conventional fluorescence imaging. Such an effort was demonstrated in [4], in which a side-viewing graded-index (GRIN) probe was inserted into the microdevice. This system demonstrated monitoring of cell apoptosis in parallel for multiple drugs released into a tumor at different locations. However, the imaging system applied was incapable of optical sectioning, the probe was rigid, and the length was on the order of only 1 cm. An advanced version would require an optical probe with three-dimensional (3D) imaging capability implemented by, e.g., confocal [5,6,7] or multiphoton [8,9,10,11] configuration. Two-photon imaging will be discussed in this work. For clinical translation of drug-response imaging in vivo, one would desire a long and flexible GRIN-lens-based microendoscope, so that the distal end of the lens may be extended from the microscope to the patient. Here, we want to mention that while GRIN multimode optical fibers nominally have the same parabolic profile as GRIN lenses, GRIN lenses usually have a more faithful parabolic index profile than GRIN optical fibers [12], possibly because of the different fabrication methods and accuracy. Therefore, in this work, the term of long GRIN lenses instead of traditional GRIN optical fibers is used to reflect this difference.

**Figure 2:**
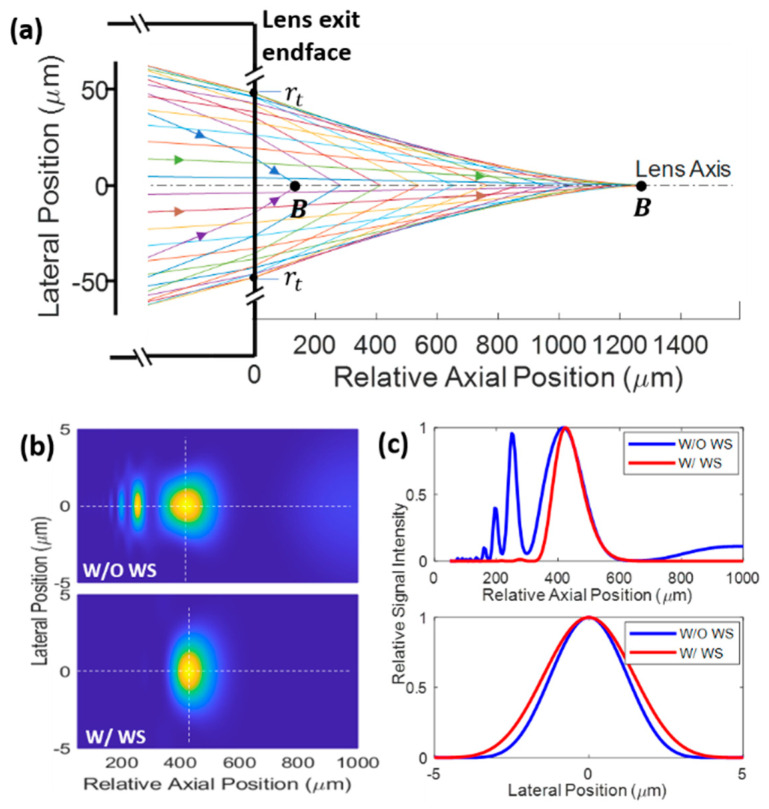
Simulated ray trajectories (**a**), intensity squared pattern $I_{f}$ (**b**), and the intensity profile (**c**) along the while dotted lines in (**b**). Simulation parameters $Z_{0}=705\;\mathrm{mm}$, ρ=250 μm, NA = 0.1, $n_{co}=1.5$, $n_{m}=1$, λ=1040 nm and f=450 μm are shown in (**c**); see main text for all the definitions. Definition of point B in (**a**) is the same as Figure 3. WS, wavefront shaping.

However, commercially available GRIN lenses are usually rigid and short (typically a few cm). The nonaplanatic property of GRIN lenses that accumulates with length is one contributor to the lens aberration. For two-photon imaging, dispersion of the lens material may lead to further aberration. These aberration sources rationalize the short length of commercial GRIN lenses. In [11], customized GRIN lenses with lengths up to 28.5 cm were investigated for clinical applications in which access to deep tissue; e.g., prostate, is necessary. For such a long and rigid lens, the axial resolution degrades significantly, but the lateral resolution does not deteriorate much. If lens length is increased further, the rays may become severely mismatched axially, as shown in Figure 2a. The intensity squared pattern (for two-photon imaging) with multiple peaks shown in the top panel of Figure 2b is a result of this mismatch. As a comparison, an ideal case would be a diffraction-limited pattern with a single peak, as shown in the bottom panel of Figure 2b, which is achievable with the wavefront shaping described later in this work. The top panel of Figure 2c suggests that the wavefront shaping is very effective in cleaning up the out-of-focus fields. However, since the GRIN lens does not introduce lateral mismatch, the field is very well confined laterally even without wavefront shaping (see the bottom panel of Figure 2c). Note that dispersion of the lens material is not included in the simulation, which may lead to experimental resolutions worse than the theoretical results shown here, but chirped mirrors may be used to alleviate the dispersion effect. In addition, note that the slightly wider lateral distribution after wavefront shaping is due to the small imaging NA (iNA). The lateral resolution is improved if a larger iNA is obtained with a shorter working distance (WD), which will be demonstrated in detail later in Section 3.

In this work, we theoretically investigate long GRIN lenses, and derive the phase compensation needed for obtaining diffraction-limited resolution. A thin GRIN lens possesses the flexibility of a typical optical fiber endoscope because of similar material properties. In practice, to prevent the long GRIN lens from breaking, it can be protected with the same polymer coating that is used for optical fibers. In addition, propagation-invariant modes of a parabolic profile GRIN lens have been shown to be almost unaffected by bending [12]. Therefore, a long and flexible GRIN lens probe may be realizable in practice, which would enable deep organ imaging for the implantable microdevices and beyond. Note that lenses with linear index profile have been investigated [13], but they are not within the scope of this work.

## 2. Theory

**Figure 3:**
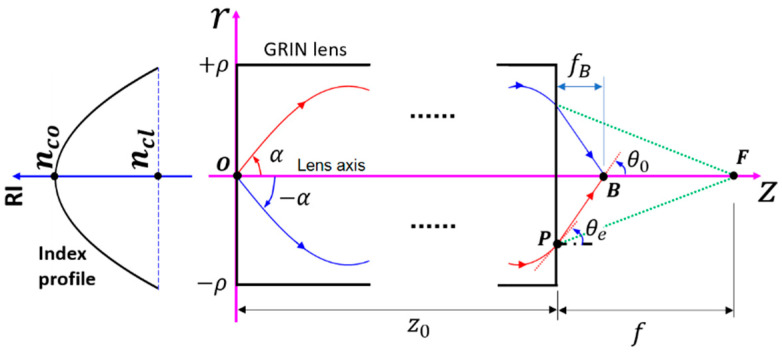
Coordinate system for describing the RI profile and ray trajectories in the meridional plane. The index profile is shown on the left.

Here, we only consider the meridional rays, using the coordinates in Figure 3. The refractive index (RI) n(r) of a parabolic profile GRIN lens in the meridional plane is expressed as:

(1) $$n^{2}\left(r\right)=\{\begin{array}{cc} n_{co}^{2}\left[1-\frac{NA^{2}}{n_{co}^{2}}{\left(r/\rho \right)}^{2}\right], & 0\leq \left|r\right|<\rho \\ n_{cl}^{2}, & \left|r\right|\geq \rho \end{array}$$

where $n_{co}$ is the RI on the axis of the GRIN lens, ρ is the lens radius, $NA=\sqrt{n_{co}^{2}-n_{cl}^{2}}$ is the lens numerical aperture (NA) with $n_{cl}$ being the cladding RI, and r is the lateral position. Note that Equation (1) is different from the conventional definition in which r is the radial position, and never takes negative values [12,14]. The lateral position r in Equation (1) may take negative values, which facilitates the description of the ray trajectories. Except for this difference, the RI profile defined in Equation (1) is identical to the conventional definition. A meridional ray trajectory associated with the ray invariant β follows the function [14]:

(2) $$r=r_{tp}\sin\left(Cz\right)$$

where

(3) $$C=NA/\left(\rho \beta \right);r_{tp}=\rho \sqrt{n_{co}^{2}-\beta^{2}}/NA$$

Note that $n_{cl}\leq \beta \leq n_{co}$ holds for bound rays, which is related to the inclination angle α (see Figure 3) through:

(4) $$\beta =n_{co}\cos\alpha$$

For bound rays, $0\leq \alpha \leq \alpha_{m}=cos^{-1}\left(n_{cl}/n_{co}\right)$. The half-period $Z_{p}$ along the axial direction is given by [14]:

(5) $$Z_{p}=\pi \rho \beta /NA$$

The optical path length along a ray trajectory is determined by the following integral:

(6) $$L_{OP}=\int_{0}^{z_{0}}\frac{n^{2}\left(r\right)}{\beta }dz=\frac{n_{co}^{2}+\beta^{2}}{2\beta }z_{0}+\frac{n_{co}^{2}-\beta^{2}}{4NA}\rho \sin\left(2Cz_{0}\right)$$

where $z_{0}$ is the lens length. The lateral position $r_{e}$ of the exit point P (see Figure 3) of a ray is:

(7) $$r_{e}=r_{tp}\sin\left(Cz_{0}\right)$$

**Figure 4:**
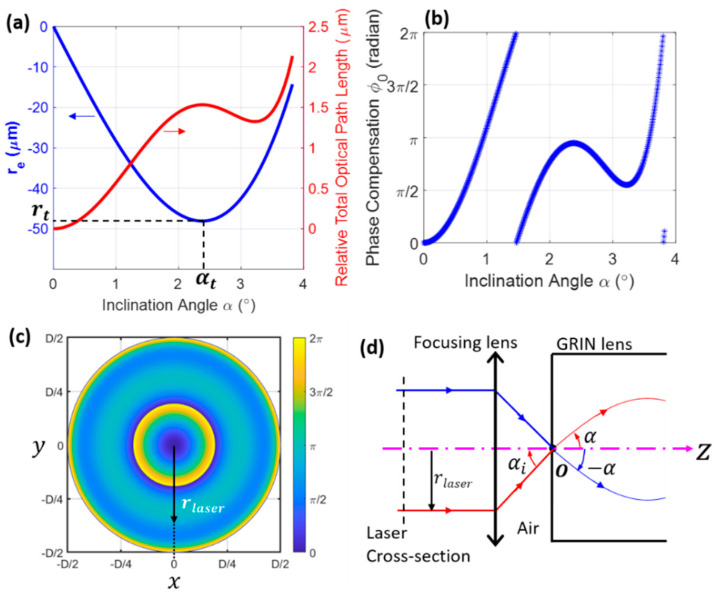
Simulated phase compensation. $r_{e}$ and relative optical path difference (**a**) and phase compensation (**b**) as a function of α. (**c**) Simulated phase compensation along the laser beam cross-section (vertical dashed line in (**d**)), which is needed for obtaining the pattern shown in the bottom panel of Figure 2b. (**d**) Schematic showing the radial position of the incident laser beam, $r_{laser}$, corresponding to a ray with inclination angle α. In (**c**), D is the laser beam diameter. Simulation parameters are identical to those in Figure 2.

For a given lens length $z_{0}$, the distance between the exit point P and the lens axis; i.e., $\left|r_{e}\right|$, may increase and then decrease as α increases (see the blue curve in Figure 4a). To calculate the turning inclination angle $\alpha_{t}$, one can take the derivative of Equation (7); i.e.,:

(8) $$\frac{dr_{e}}{d\alpha }=\frac{\rho n_{co}}{NA}\cos\alpha \sin\left(Cz_{0}\right)+z_{0}\tan^{2}\alpha \cos\left(Cz_{0}\right)$$

and thus $\alpha_{t}$ is the solution when Equation (8) is equal to zero. The turning lateral position $r_{t}$ is obtained by inserting $\alpha_{t}$ into Equation (7). At the exit point P, the incident angle $\theta_{e}$ on the lens side is made by the tangent line of the trajectory with the positive z axis (Figure 3), which satisfies:

(9) $$\tan\theta_{e}={\frac{dr}{dz}|}_{z_{0}}=r_{tp}C\cos\left(Cz_{0}\right)$$

Using Snell’s law, the refraction angle $\theta_{0}$ when the ray exits the GRIN lens is governed by:

(10) $$n\left(r_{e}\right)\sin\theta_{e}=n_{m}\sin\theta_{0}$$

where $n_{m}$ is the RI of the surrounding medium, which is typically air or water. The refractive straight ray is governed by the following equation:

(11) $$r=r_{e}+\tan\theta_{0}\left(z-z_{0}\right)$$

If B is defined as the point of intersection between the refractive ray and lens axis, then the axial distance $f_{B}$ between B and the lens exit end face (See Figure 3) is given by:

(12) $$f_{B}=-r_{e}/\tan\theta_{0}$$

Since $f_{B}$ is dependent on the inclination angle α, all the rays intersect with the lens axis at different positions, as demonstrated in Figure 2a, which results in the multiple-peak pattern in Figure 2b. To form a single-peak pattern, like the one in Figure 2c, one may modify the phase of the incident rays. To do this, we first define a point F on the lens axis, which is here called the design focal point, around which the single-peak pattern appears. Then, the total optical path length for a ray travelling from the starting point O to F is determined by:

(13) $$L_{total}=L_{OP}+L_{PF}$$

where

(14) $$L_{PF}=n_{m}\sqrt{r_{e}^{2}+f^{2}}$$

where f is the axial distance between point F and the lens exit endface (see Figure 3), which here is called the design WD. The red curve in Figure 4a shows the optical path difference (OPD) as a function of the incident angle when f=450 μm. To compensate for the OPD, an initial phase $\phi_{0}$ can be introduced to the incident rays, which is given by:

(15) $$\phi_{0}\left(\alpha \right)=\left(L_{total}-{L_{total}|}_{\alpha =0}\right)k$$

where k=2π/λ is the vacuum wavenumber, with λ being the wavelength in vacuum. Figure 4b shows such an initial phase as a function of the inclination angle. With this phase compensation, all the rays are in phase when they reach the designed focal point F. In practice, the initial phase of the incident laser beam can be manipulated via a spatial phase modulator [15], deformable mirror [5], or liquid crystal [16], following the phase pattern shown in Figure 4c. In addition, note that another way to modify the phase is through microstructures constructed on the proximal endface of the lens [17,18]. According to the geometric relations shown in Figure 4d and the Fresnel laws, the radial position $r_{laser}$ in Figure 4c is proportional to $tan\left[sin^{-1}\left(n_{co}sin\alpha \right)\right]$. With this phase modulation, the exact interference pattern shown in the bottom panel of Figure 2b is achieved.

**Figure 5:**
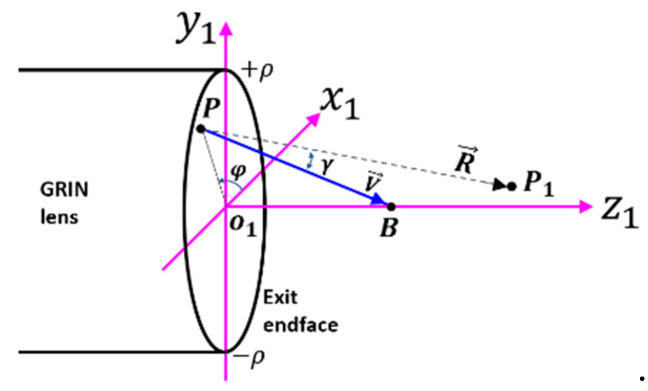
Coordinate system for calculating the diffraction pattern. Definitions of P and B are the same as those in Figure 3.

With all the ray parameters being determined, following the coordinates in Figure 5, the interference pattern around the design focal region is simulated by the first Rayleigh–Sommerfeld diffraction integral [19]:

(16) $$U\left(P_{1}\right)=-\frac{j}{\lambda }\iint_{\sum }U(P)\frac{e^{-jk_{m}R}}{R}\cos\left(\vec{V},\vec{R}\right)ds$$

where $P_{1}$ and P are a point around the focal region and source region ∑, respectively; U is the light field amplitude; j is the imaginary unit; $k_{m}=kn_{m}$ is the wavenumber in the surrounding medium; $\vec{R}$ is the directional vector pointing from P toward $P_{1}$, and $\vec{V}$ is the directional vector pointing from P toward B. To apply Equation (16), one should note that the source region ∑ has an overlapped region when there is a turning angle $\alpha_{t}$. For convenience, Equation (16) is rewritten in terms of the azimuthal angle φ and inclination angle α; i.e.,:

(17) $$U\left(P_{1}\right)=-\frac{j}{\lambda }\times \int_{0}^{2\pi }d\varphi \int_{0}^{\alpha_{m}}E\left(\alpha \right)e^{j\phi_{0}\left(\alpha \right)}e^{-jkL_{OP}}\frac{e^{-jk_{m}R}}{R}\cos\gamma \left|r_{e}\right|\left|\frac{dr_{e}}{d\alpha }\right|d\alpha$$

where $dr_{e}/d\alpha$ is given by Equation (8), γ is the angle made by $\vec{V}$ and $\vec{R}$, and E(α) is the field distribution of the source, which is assumed to be unity in all calculations. By resorting to the Cartesian coordinate in Figure 5, vectors $\vec{V}$ and $\vec{R}$ are expressed as:

(18) $$\left\{ \begin{array}{l} \vec{V}=\left(-\left|r_{e}\right|\cos\varphi ,-\left|r_{e}\right|\sin\varphi ,f_{B}\right)\\ \vec{R}=\left(x_{1}-\left|r_{e}\right|\cos\varphi ,y_{1}-\left|r_{e}\right|\sin\varphi ,z_{1}\right) \end{array}\right.$$

where $\left(x_{1},y_{1},z_{1}\right)$ are the Cartesian coordinates of $P_{1}$. Then:

(19) $$\cos\gamma =\frac{\vec{V}\cdot \vec{R}}{VR}$$

Note that V and R are the lengths of $\vec{V}$ and $\vec{R}$, respectively. Since we are interested in two-photon imaging, the fluorescent signal intensity $I_{f}$ is proportional to the excitation intensity squared [20], then we have:

(20) $$I_{f}\propto {\left|U\right|}^{4}$$

## 3. Results and Discussion

**Figure 6:**
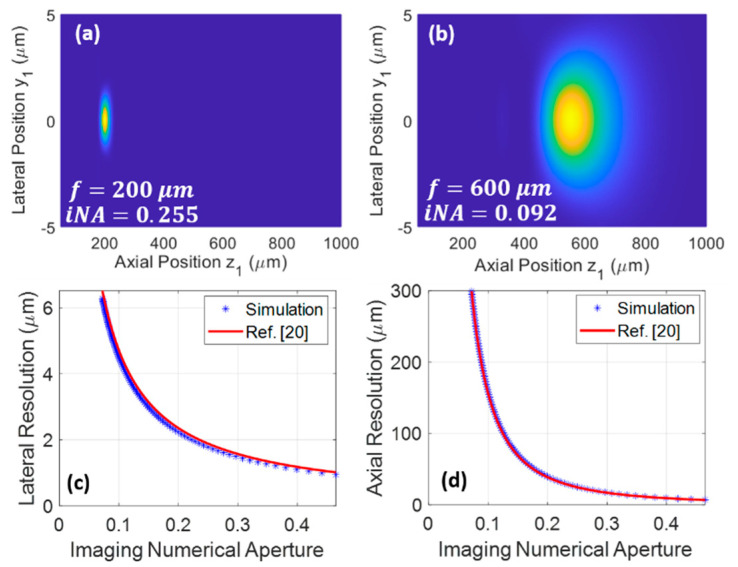
Simulated resolution. Distribution of $I_{f}$ when f=200 μm (**a**) and 600 μm (**b**). Calculated lateral (**c**) and axial (**d**) resolution vs. imaging numerical aperture (iNA). Here, resolution is defined by the 1/e full width of the signal intensity (i.e., $I_{f}$) fitted to a Gaussian function. Simulation parameters are the same as those in Figure 2, except $Z_{0}=1058\;\mathrm{mm}$ (equal to 90 times average $Z_{p}$ minus 1 mm).

Using the above theory, performance of the wavefront shaping for improving the resolution is readily evaluated. As two examples showing the diffraction pattern at different design WDs, Figure 6a,b display the pattern when f=200 μm and f=600 μm, respectively. The light field is confined to a much tighter spot in Figure 6a, which is due to the larger iNA. Here, note that the iNA is different from the NA of the GRIN lens defined in Equation (1); it takes the following definition:

(21) $$iNA=n_{m}\sin\left[\tan^{-1}\left(\left|r_{t}\right|/f_{m}\right)\right]$$

where $f_{m}$ is the axial distance between the point with the highest intensity and the lens exit endface, and $r_{t}$ is the aforementioned turning lateral position. Note that $f_{m}$ is generally a little smaller than the design focal length f, mainly because the field amplitude is inversely proportional to the source-image distance R.

The blue dotted lines in Figure 6c,d show the simulated lateral and axial resolution as a function of iNA. For comparison, the diffraction-limited lateral and axial resolutions computed from the equations in Figure 4c of [20] are shown as the red curves. The very close agreement between these curves suggests that the lens reaches the diffraction-limited resolution after the wavefront shaping. Note that the resolution is defined as the full width at the 1/e signal intensity level after the simulated data is fitted to a Gaussian function. We want to emphasize that these are on-axis resolutions; the off-axis resolutions are expected to degrade gradually as the off-axis distance increases. Resolution defined by the full width at half maximum can be calculated by the 1/e resolution divided by $\sqrt{ln2}$. It is also noteworthy that, although a GRIN lens with low NA of 0.1 is used in the simulations, iNA of over 0.4 is achievable for high-resolution imaging. This singlet configuration may reduce the cost required by conventional doublets consisting of a low-NA relay lens and a high-NA imaging lens, or triplets consisting of a low-NA relay lens and two high-NA imaging lenses on both ends for coupling and imaging.

Wavefront shaping has been extensively used in experiments to manipulate the focal point out of multimode fibers [21,22,23,24,25]. Those methods include a complicated calibration process that requires access to the distal end of the probe, except for the case in which a thin stack of structured metasurface reflectors is used for feedback [26]. In our work, the compensation phase is predetermined analytically, and thus no calibration process is needed. Then, the 3D imaging can be realized by galvanometer mirrors in combination with a tunable lens [9] or a translational stage [8].

## 4. Conclusions

In conclusion, we have demonstrated analytically that diffraction-limited imaging through long-GRIN-lens-based microendoscopes is made possible by wavefront shaping. Taking advantage of the available analytical solution of the propagation modes in GRIN lenses with a parabolic index profile, the phase difference between different modes can be determined analytically. Diffraction-limited resolution is then obtainable after the phase difference is corrected. In practice, the phase difference can be compensated by using a spatial light modulator or other techniques. It was also found that, using a short design WD, a low-NA GRIN lens may be used as a high-resolution probe with a large iNA; this singlet configuration will significantly simplify the traditional design of doublets or triplets with equivalent iNA. In future experiments, a long sub-millimeter-diameter GRIN lens with protective coatings may serve as a promising flexible probe for the emerging microdevice to be implanted in deep tissues.

## Data Availability

The data presented in this study are available on request from the corresponding author.
